# Effects of Light Intensity Activity on CVD Risk Factors: A Systematic Review of Intervention Studies

**DOI:** 10.1155/2015/596367

**Published:** 2015-10-12

**Authors:** Romeo B. Batacan, Mitch J. Duncan, Vincent J. Dalbo, Patrick S. Tucker, Andrew S. Fenning

**Affiliations:** ^1^Institute for Health and Social Science Research, Central Queensland University, Bruce Highway, Rockhampton, QLD 4702, Australia; ^2^Centre for Physical Activity Studies, Central Queensland University, Bruce Highway, Rockhampton, QLD 4702, Australia; ^3^School of Medicine & Public Health, Priority Research Centre for Physical Activity and Nutrition, Faculty of Health and Medicine, The University of Newcastle, University Drive, Callaghan, NSW 2308, Australia; ^4^Clinical Biochemistry Laboratory, Central Queensland University, Bruce Highway, Rockhampton, QLD 4702, Australia

## Abstract

The effects of light intensity physical activity (LIPA) on cardiovascular disease (CVD) risk factors remain to be established. This review summarizes the effects of LIPA on CVD risk factors and CVD-related markers in adults. A systematic search of four electronic databases (PubMed, Academic Search Complete, SPORTDiscus, and CINAHL) examining LIPA and CVD risk factors (body composition, blood pressure, glucose, insulin, glycosylated hemoglobin, and lipid profile) and CVD-related markers (maximal oxygen uptake, heart rate, C-reactive protein, interleukin-6, tumor necrosis factor-alpha, and tumor necrosis factor receptors 1 and 2) published between 1970 and 2015 was performed on 15 March 2015. A total of 33 intervention studies examining the effect of LIPA on CVD risk factors and markers were included in this review. Results indicated that LIPA did not improve CVD risk factors and CVD-related markers in healthy individuals. LIPA was found to improve systolic and diastolic blood pressure in physically inactive populations with a medical condition. Reviewed studies show little support for the role of LIPA to reduce CVD risk factors. Many of the included studies were of low to fair study quality and used low doses of LIPA. Further studies are needed to establish the value of LIPA in reducing CVD risk.

## 1. Introduction

Cardiovascular disease (CVD) remains the leading cause of death worldwide [1]. Several biological risk factors, such as male gender, family history of heart disease, high blood pressure (BP), dyslipidemia, obesity, glucose abnormalities, insulin resistance, and lifestyle risk factors, such as smoking, poor diet, lack of physical activity, low cardiorespiratory fitness, excessive alcohol use, and stress, are associated with the development and progression of CVD [2, 3]. Notably, these lifestyle risk factors strongly influence the established biological CVD risk factors and also affect novel pathways of risk such as inflammation [4]. For instance, physical activity and cardiorespiratory fitness (measured by maximal oxygen consumption (VO_2_ max) and heart rate (HR)) are known to improve a number of traditional biological risk factors for CVD, including BP [5], high-density lipoprotein (HDL) cholesterol [6], body fat [7], and novel risk factors such as C-reactive protein (CRP) levels [8].

There is excellent evidence that physical activity, particularly moderate-to-vigorous intensity physical activity (MVPA), is effective in the prevention and treatment of CVD [9, 10]. The existing public health guidelines emphasize participation in MVPA to achieve health benefits [9, 10]. However, the view that physical activity has to be moderate to vigorous to achieve cardiovascular risk reduction has been questioned [11]. It is suggested that physical activity performed at light intensity level can also provide health benefits [12, 13]. As such, although early studies demonstrate that light intensity physical activity (LIPA) (20 < 40% VO_2_ max [14]) is not associated with reduced CVD and overall mortality rates [15, 16], there is growing recognition of the potential for LIPA to reduce disease risk, particularly CVD [17]. This is emphasized by cross-sectional studies demonstrating that LIPA is associated with CVD risk factors [12, 13, 18]. LIPA is important to understand, from a health perspective, as adults tend to spend a greater portion of their day (6.5 hr/day [13, 14]) performing LIPA compared to MVPA (0.7 hr/day [13, 14]). Many people often find it more attractive and attainable to perform LIPA than MVPA (40 < 85% VO_2_ max) [19]. Furthermore, recent evidence suggests that muscle fiber recruitment during LIPA may potentially produce cellular signals which may regulate risk factors for disease [20].

As a result, clarifying the role of LIPA in CVD prevention is important given the amount of time people spend engaged in light intensity activities and its potential as an intervention target. To date, there has been no comprehensive review of literature describing the role of LIPA on CVD risk factors. Therefore, the aim of this review is to systematically examine the effects of LIPA on CVD risk factors (body composition, BP, glucose, insulin, glycosylated hemoglobin, total cholesterol, low-density lipoprotein (LDL) cholesterol, HDL cholesterol, and triglycerides) and other CVD-related markers (VO_2_ max, HR, CRP, interleukin-6, tumor necrosis factor- (TNF-) alpha, TNF receptor 1 (TNFR1), and TNF receptor 2 (TNFR2)) in adults.

## 2. Methods

**Figure 1:**
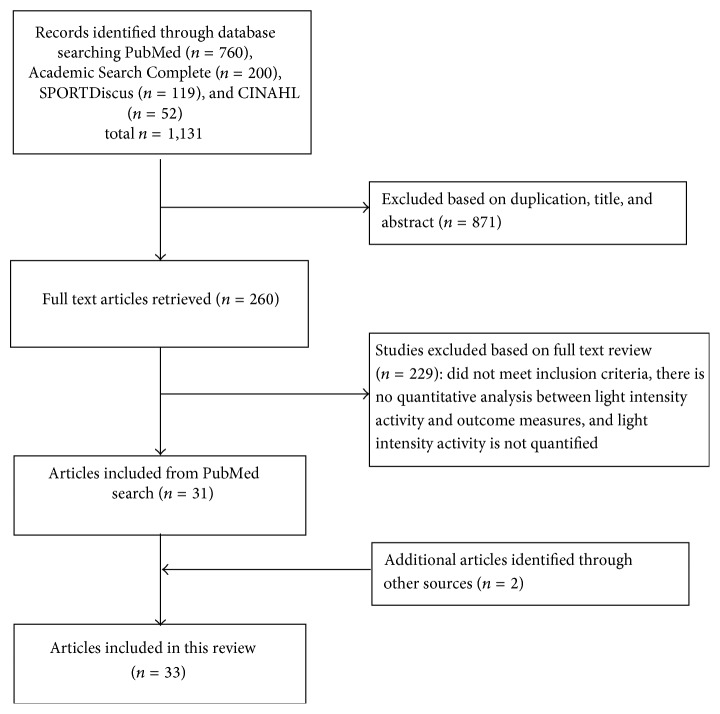
Flow diagram of study selection.

A systematic search was performed on 15 March 2015 according to PRISMA guidelines [21]. Articles were retrieved from PubMed, Academic Search Complete, SPORTDiscus, and CINAHL using multiple search criteria provided in Supplementary Table  1 in Supplementary Material available online at http://dx.doi.org/10.1155/2015/596367. Initially, titles and abstracts of identified articles were checked for relevance by two reviewers (RB and PT). Subsequently, both reviewers independently reviewed the full text of potentially eligible papers. Any disagreement between the two reviewers for inclusion was resolved through discussion. Additional articles were identified via hand-searching and reviewing the reference lists of relevant papers.  Figure 1 presents the flow of papers through the study selection process.

Studies were considered to be eligible for inclusion based on the following criteria: (i) participants were ≥ 18 years of age; (ii) the study examined at least one of the following CVD risk factors/markers in humans: body mass, body mass index (BMI), waist circumference (WC), hip circumference, waist-to-hip ratio (WHR), % body fat, HR, BP, VO_2_ max, glucose (fasting or postprandial), glycosylated hemoglobin, insulin, total cholesterol, HDL cholesterol, LDL cholesterol, triglycerides, CRP, interleukin-6, TNF-alpha, TNF receptor 1, or TNF receptor 2 levels; (iii) the study reported an intervention (both randomized and nonrandomized) that imposed on participants a single or periodic bouts of LIPA defined as activities between 1.6 < 3.0 METs, 20 < 40% VO_2_ max, and 20 < 40% heart rate reserve (HRR) or the relative intensity of 40 < 55% HR max [14, 22]; (iv) the study included quantitative analysis (statistical comparison of intervention to baseline or a control group) of the effect of LIPA on at least one of the outcome measures; (v) the study was published or accepted for publication in refereed journals from 1970 up to and including the search date; (vi) the study was published in the English language. Due to the lack of a standardized definition of LIPA for resistance training, only aerobic/flexibility exercises were included in the study.

Two authors (RB and PT) independently assessed the quality of the studies that met the inclusion criteria (Table 1). The risk of bias and strength of evidence from individual studies were assessed using Downs and Black Checklist [23], allowing for the assessment of the methodological quality of randomized controlled trials and nonrandomized studies of health care interventions. This 27-point checklist assesses the strength of reporting, external validity, internal validity, and statistical power. As some questions are worth more than one point, the maximum score that can be received is 32. Adapted from another systematic review [24], the score obtained by each study was divided by 32 and multiplied by 100 to provide a “study quality percentage.” Study quality percentages were then classified as high (66.7% or higher), fair (between 50.0 and 66.6%), and low (less than 50.0%) [24].

Following data extraction, the interventions included in this review were heterogeneous in terms of the type, frequency, and duration of physical activities, as well as body mass, physical fitness, and dietary intake of the participants. Thus, meta-analyses or pooling of data across studies would be inappropriate so a qualitative synthesis of the evidence was performed instead.

A modified form of coding system described by Sallis et al. [25] was used to summarize the effect of LIPA on CVD risk factors/markers. If 0–33% of the studies reported a statistically significant difference between LIPA and CVD risk factors/markers, the result was categorized as no effect (0). If 34–59% of the studies reported a statistically significant difference, the result was categorized as inconsistent (?). If 60–100% of the studies reported a statistically significant difference, the result was rated as positive (+) or negative (−), respective of the direction of the effect. When four or more studies supported a difference or no difference, it was coded as ++, − −, or 00 to indicate consistent observations. The ?? code indicated a marker that has been examined in four or more studies with inconsistent findings (e.g., out of 5 studies, 3 indicated a significant positive effect and 2 indicated a significant negative effect).

Results were then stratified by health status of the population (healthy or those with a medical condition). Studies in which participant physical activity was less than 150 min/wk of moderate intensity physical activity or 75 min/wk of vigorous intensity physical activity or participants were not engaged in regular physical activity/exercise (as described in the primary study) or participants were defined as sedentary were subsequently classified as “physically inactive” and the results are summarized separately for these studies.

## 3. Results

General study characteristics are summarized in Table 1; more detailed study characteristics are presented in Supplementary Table  2. All studies had LIPA intervention with the number of study participants in the LIPA group ranging from *n* = 6–39. Participants were primarily young, adult, and males (18–39 years old). Duration of LIPA interventions ranged from an acute bout of training lasting 5 minutes to chronic training lasting 30 min per session, 3 times per week, for 9 months. The exercise modalities involved stretching and calisthenics (*n* = 1), hand rim wheelchair training (*n* = 1), yoga (*n* = 1), standing (*n* = 2), slow flexibility exercises (*n* = 3), stationary cycling (*n* = 7), and slow home/outdoor or treadmill walking (*n* = 18). Fifteen [26–40] of the 33 reviewed studies had participants classified as overweight or obese and 7 studies [31, 33, 34, 36, 41–43] consisted of participants suffering from medical conditions (hypertension, diabetes mellitus, chronic heart failure, gestational diabetes, colorectal cancer, metabolic syndrome, and HIV infection).

Studies included in this review were assigned a quality rating. The study quality was rated low in 11 studies, fair in 20 studies, and high in 2 studies. Over 90% of the studies scored 1 point for defining study objectives, describing exposure and outcome variables, utilizing random sampling of the target population, providing data sources, and describing the data collection process. Seventeen studies (51%) [26, 34, 36, 39, 42, 44–55] did not describe participant recruitment methods and 10 studies (30%) [26, 40, 44, 46–52] did not report inclusion criteria. Five studies (15%) [33, 34, 41, 56] did not report compliance rates. Of the studies that reported compliance, compliance was generally good with participants completing 81% to 100% of the activity sessions implemented per study design. Six studies [26, 31, 35, 38–40] reported power calculations relevant to their study aims. All but 4 studies [26, 30, 31, 33] performed the activity sessions in a laboratory/clinic directly supervised by a research staff. Those not conducted in the laboratory, light intensity activity was performed outdoors/at home with participants monitoring their own HR.

A summary table of the effect of LIPA on CVD risk factors and markers can be found in Table 2; the effects of LIPA on CVD risk factors/markers reported in each study are presented in Supplementary Table  3. Results demonstrated LIPA training interventions to have no significant effect on markers of body composition in physically inactive or healthy, with a medical condition, adults. All studies that examined the effect of LIPA on body mass [26, 29, 35, 53], WC [31, 32, 35, 41], BMI [26, 31, 32, 41], and % body fat [30, 32] reported no significant change. LIPA was found to have no effect on systolic or diastolic BP in healthy adults while improvements in BP were found in physically inactive populations with a medical condition. Three [31, 32, 39] of 9 studies (33%) reported significant decreases in systolic BP while 2 [31, 39] of 9 studies (22%) reported significant decreases in diastolic BP.

LIPA was found to have no effect on glucose and insulin response in physically inactive or healthy, with a medical condition, adults. Three [33, 38, 40] of 16 studies (19%) reported significant decreases in glucose and 1 of 13 (8%) reported significant decrease in insulin level. When the effect of LIPA on blood lipid markers was examined, no significant changes were found for total cholesterol, HDL cholesterol, LDL cholesterol, or triglycerides in physically inactive or healthy individuals and inconsistent findings on triglycerides in healthy adults. One [50] of 11 studies (9%) reported a significant increase in total cholesterol while 2 [26, 46] of 11 studies (18%) reported significant decreases in total cholesterol. One [50] of 13 studies (8%) reported a significant increase in HDL cholesterol, 5 [36, 46, 49, 54, 57] of 13 studies (38%) reported significant increases in triglycerides, and 0 of 6 studies (0%) reported an effect on LDL cholesterol.

Regarding other CVD-related markers, the effect of LIPA on VO_2_ max is inconclusive in physically inactive or healthy adults. Three [27, 32, 56] of 8 studies (38%) reported significant increases in VO_2_ max. LIPA was also found to have no effect on resting HR in physically inactive or healthy adults. One [32] of 5 studies (20%) reported a significant reduction in resting HR. All studies that examined the effect of LIPA on CRP [30, 37], interleukin-6 [30, 34, 37, 43], and TNF-alpha [30, 34] reported no significant effect in physically inactive, with a medical condition, adults.

## 4. Discussion

The effect of LIPA on markers of cardiovascular risk factors was systematically reviewed. LIPA resulted in no significant improvements in body composition, glucose, insulin (in physically inactive or healthy, with a medical condition, adults), total cholesterol, HDL cholesterol, LDL cholesterol (in physically inactive or healthy adults), or triglycerides (in physically inactive adults) and inconsistent findings on triglycerides in healthy adults. On the other hand, LIPA was found to improve systolic and diastolic BP in physically inactive populations with a medical condition. Additionally, when examining CVD-related markers, we found inconsistent results regarding the effect of LIPA on VO_2_ max in physically inactive or healthy adults, no significant changes on resting HR in physically inactive or healthy adults, and no significant changes on inflammatory markers in physically inactive or with a medical condition adults.

Nine studies [26, 29–32, 35, 41, 53, 55] examined the effect of LIPA on body composition and found no effect in either physically inactive or healthy, with a medical condition, populations. One study concluded that LIPA performed 30 min, 8 times a day, for 5 days, did not result in any significant change on body mass and WC [35]. The rest of the studies demonstrated that LIPA performed 30–90 min, 3 to 5 times per wk, for ≥7 wk, did not result in any significant effect on body mass [26, 29, 53, 55], WC [31, 32, 41], BMI [26, 31, 32, 41], WHR [26], or % body fat [30, 32]. This result is consistent with previous research findings that conclude at least 250 min/wk of moderate intensity (≥3 METs) training is needed if the primary purpose of the training program is to elicit reductions in body mass and fat mass [58, 59]. There are no recommended durations of LIPA required to elicit weight loss; however, the amount of LIPA required to improve body composition is likely to be much greater than that required for MVPA given the reduced intensity level. Physical activity alone if greater than 250 min/wk without caloric restriction has a limited influence on body composition [27, 28, 60] and may only cause 1–3% change in body mass and adipose tissue [61]. In addition, evidence suggests that the total volume of physical activity is a key factor in achieving weight loss [62]. An individual intending to lose weight through physical activity without dietary restriction would need to engage in a large volume (26 MET-hr per wk) of physical activity to achieve a 5% weight reduction [62]. In most studies to date, the volume of LIPA used is less (<10.5 MET-hr per wk) than the 26 MET-hr per wk that may be required to improve body composition.

Findings of this review also indicate no significant changes in resting BP in healthy adults but found significant improvements in physically inactive individuals with a medical condition. Participants who followed a single bout (15 min) [52] and periodic bouts (2 min every 20 min over 5 hr period) [40] of treadmill walking, and long term (30–60 min 3–5x/wk, ≥10 wk) LIPA [30, 32, 41, 53, 56] demonstrated no significant changes in resting BP. Two studies [31, 32] reported a decrease in systolic BP and one study reported [31] a decrease in diastolic BP following ≥ 10 wk of walking [31] and a combination of treadmill walking, stationary cycling and stepping [32]. In both studies, the improved BP response was found in physically inactive participants with hypertension. Similarly, in prehypertensive and hypertensive physically inactive, obese adults, one study [39] with a different study design (randomized cross-over study breaking up prolonged sitting with LIPA breaks) found significant reductions in systolic and diastolic BP in individuals interrupting sitting time with light intensity walking relative to individuals with uninterrupted sitting. Thus, LIPA appears unlikely to influence the BP response in normotensive populations but may be able to provide an effect in hypertensive, physically inactive populations.

There were no significant improvements in glucose and insulin response following LIPA in either physically inactive or healthy, with a medical condition, adults. All 6 studies [36, 44, 45, 47, 49, 63] reported no effects of glucose and insulin response during a single bout (35–237.5 min) of LIPA. Following periodic bouts (214.5 ± 28 min divided in 9 bouts, 30–60 min 3x/wk, 30 min 8x/day, 4 hr walking, and 2 hr standing/day) of LIPA, 7 [30, 32, 35, 41, 53, 54, 57] of 10 studies reported no significant changes in glucose and 6 [30, 32, 35, 53, 54, 57] of 7 studies reported no significant changes in insulin. These results are consistent with epidemiological data showing no significant association between fasting glucose and time spent performing LIPA (5.7–6.0 hr/day) (but not with 2 hr plasma glucose which was found to be significantly associated with LIPA) [12, 13]. In contrast, 2 studies [38, 40] reported a decrease in postprandial glucose (and insulin [38]) after interrupting sitting with light intensity standing/walking. These laboratory-based studies compared a light intensity standing/walking group to a sitting group, employed LIPA (14 sessions of 2 min LIPA separated by 20 min sitting period) dispersed throughout the day, and measured postprandial glucose. These findings were validated in a recent meta-analysis that found significant reductions in blood glucose postprandial response and insulin levels after interrupting sedentary periods with LIPA breaks [64]. Another study [33] with a longer, structured, light intensity walking intervention period (120–160 min/wk walking for 6 wk) also demonstrated reductions in capillary glucose concentrations post-intervention compared to baseline. This study used obese women with gestational diabetes. In summary, there is no consistent intervention evidence to support improved glucose metabolism with LIPA in healthy adults. Studies [33, 38, 40] suggesting that LIPA may improve glucose and insulin response examined individuals with higher glucose baseline values or compared LIPA to a sedentary (sitting) group or used multiple bouts of LIPA dispersed throughout the day. Thus, there may be some evidence to support the view that LIPA influences glucose and insulin metabolism, but this evidence appears to be limited to individuals (1) with impaired cardiometabolic function or (2) who are compared to no activity (sedentary) control groups.

Most studies in this review reported no significant change in total cholesterol [30, 32, 40, 47, 49, 51, 53, 57], HDL cholesterol [26, 30, 32, 36, 40, 46, 47, 49, 51, 53, 57, 63], LDL cholesterol [26, 30, 32, 46, 51, 57], in physically inactive or healthy individuals, or in triglycerides [26, 30, 32, 35, 40, 51, 53, 63] in physically inactive adults following LIPA. Inconsistent findings were found on the effect of LIPA on triglycerides in healthy adults. This result is not consistent with evidence from epidemiological studies demonstrating a beneficial association of larger volumes of LIPA with HDL cholesterol (4.3 hr/day of LIPA) [65] and triglycerides (5.7–6.0 hr/day; <150 min/wk MVPA, but LIPA exceeded sedentary behavior) [18, 66]. Conversely, similar results were found in a recent meta-analysis that reported no significant reductions in triglycerides after interrupting sedentary periods with LIPA breaks [64]. These conflicting results reported in the literature in regard to the effect of LIPA in blood lipid markers may be due to differences in study protocol and participants' baseline lipid levels.

In this review, 5 [36, 46, 49, 54, 57] of 10 studies demonstrated significant reductions in triglycerides following LIPA in healthy adults. These studies used short intervention periods (≤4 days) of light intensity walking and 3 studies [36, 49, 54] used a high fat test meal prior to blood sampling. This immediate lowering of serum triglycerides following LIPA is most likely due to enhanced triglyceride peripheral tissue uptake of serum triglycerides that result from exercise-induced activity of lipoprotein lipase, the rate limiting enzyme for the hydrolysis of triglyceride-rich lipoproteins [67]. The increased activity of lipoprotein lipase (persisting up to 18 hr) following muscular contractions causes an increase in the removal of triglycerides from the circulation [68]. Unfortunately, none of these studies explored whether or not triglyceride reductions persisted for more than 24 hr following LIPA bout.

VO_2_ max, resting HR, and inflammatory markers that are known to impact CVD risk factors were also examined. LIPA had inconsistent results in regard to the effects on VO_2_ max and no effect on resting HR in physically inactive or healthy adults. Studies employing long term (≥8 wk) LIPA protocols generally reported no change [28–30, 42, 48, 53, 56], while others reported improvement in VO_2_ max [27, 32, 56] and HR [32]. It is possible that certain types of aerobic exercise may lead to health-related benefits and yet may not be of sufficient quantity or quality to improve VO_2_ max or decrease resting HR [69]. Despres and Lamarche [70] proposed that prolonged (exact duration not specified) low intensity (approximately 50% VO_2_ max) endurance exercise performed 45–60 min on an almost daily basis significantly improved insulin sensitivity and lipoprotein metabolism through mechanisms that are likely to be independent of the training-related changes in cardiorespiratory fitness. The proposed mechanisms included the net increase in energy expenditure and losses in total body fat and abdominal adipose tissue which contributed to improved carbohydrate and lipid metabolism [70]. This hypothesis, however, remains to be established. At present, only 3 [27, 32, 56] of 7 studies reported a positive effect on VO_2_ max; 2 [27, 32] of these 3 studies examined physically inactive, overweight adults. The third study [56] neglected to report baseline physical activity and BMI. Thus, the beneficial effects of LIPA, in regard to adaptations to VO_2_ max, are equivocal and may be most pronounced in individuals with low levels of physical activity [71, 72] suggesting that the benefits of LIPA on VO_2_ max may be limited to populations who are least active.

No significant changes in inflammatory markers (CRP, interleukin-6, and TNF-alpha) were found in physically inactive or with a medical condition participants engaging in a single bout (40–60 min) [37, 43], periodic bouts (40 min/day for two wk) [34], or long term (30 min 3x/wk for 16 wk) [30] LIPA. Research in this area is limited and more studies are needed to clarify the effect of LIPA on inflammatory markers. Results from the interventions (3 out of 4 studies) included in this review demonstrate that acute effects are unlikely to occur and future research should seek to examine changes in inflammatory markers following participation in LIPA over longer time periods.

This review provides consistent evidence that LIPA is not effective at improving CVD risk factors and other CVD-related health markers in apparently healthy individuals. Some evidence surfaced suggesting that LIPA may improve markers of CVD risk factors (BP) in physically inactive adults with a medical condition. These findings provide some support to cross-sectional studies suggesting that LIPA may be beneficial in elderly, physically inactive, with a medical condition, individuals [65, 73, 74]. However, due to limited intervention studies available that have examined these cohorts of individuals, it is difficult to make conclusions with full certainty. Future studies should attempt to elucidate the effects of LIPA in elderly, physically inactive, with a medical condition, adults. Since LIPA is low intensity and appears to be most practical in physically inactive populations, daily LIPA and MVPA of participants should be accounted for in future work.

The dose of LIPA used in the reviewed studies was modest in comparison to the volume of LIPA typically performed by individuals (e.g., ≤150 min/wk which equates to <10.5 MET-hr per wk). Therefore, future studies are encouraged to use greater doses (much higher than the recommended 150 min/wk moderate intensity physical activity due to the reduced intensity level of LIPA) to assist in clarifying the role of LIPA to elicit positive changes in CVD risk factors and CVD-related markers.

## 5. Conclusions

Although cross-sectional research findings [12, 13, 18] suggest that LIPA may help to improve an individual's metabolic profile, there is no evidence to support the effect of LIPA in providing positive changes in CVD risk factors in healthy adults. Little intervention evidence was found to support the positive effect of LIPA in CVD risk factors in physically inactive adults with a medical condition. In particular, significant improvements in BP following LIPA were achieved by physically inactive, hypertensive individuals [31, 32, 39]. However, it should be noted that many studies reviewed did not control, either statistically or by design, for potential confounding variables such as controlling for accumulated MVPA or monitoring dietary intake. Most of the studies have also used small doses of LIPA (<10.5 MET-hr per wk). Given that adults spend a considerable proportion of their day (6.5 hr/day [13, 14]) performing LIPA, it may be possible that this volume of LIPA is not enough of a stimulus to promote favorable adaptations in the examined biological markers of CVD risk. Aside from increasing the volume, it may also be worthwhile to examine the effects of LIPA dispersed throughout the day similar to recent studies [35, 38–40] that have used regular short bouts of LIPA to interrupt prolonged periods of sitting. This may be useful as recent meta-analysis found these breaks in sitting to be associated with improved glucose and insulin response [64]. In summary, there may be some evidence to support the view that LIPA influences some CVD risk factors in certain populations, but more well-designed experiments with greater control of confounding factors are required to confirm this.

## Supplementary Material

The Supplementary Material files provide the search criteria used in the review (Supplementary Table 1), detailed description of included studies (Supplementary Table 2), and the summary effects of LIPA on CVD risk factors and CVD-related markers reported per study (Supplementary Table 3).

## Figures and Tables

**Table 1:** Summary of included studies, sorted by disease status and duration of intervention.

Study reference	Disease status^c^	Duration of intervention	Weight status (based on mean BMI)	Study design	Sample size *N* = LIPA group (total)	Age range (mean ± SD)	Baseline activity level^c^	Modality of intervention	Downs and Black Score	Study quality
[56]	No	9 mo	No data	RCT	33 (72)	60–81 (63.9 ± 3.9)	No data	Laboratory-based	17	Fair
[26]^a^	No	24 wk	Overweight	RCT	12 (36)	18–45	Sedentary	Free-living	16	Fair
[30]	No	16 wk	Obese	RCT	6 (25)	(52.8 ± 7.2)	Sedentary	Free-living	19	Fair
[28]	No	16 wk	Overweight	RCT	17 (51)	20–50	Sedentary	Laboratory-based	19	Fair
[27]	No	16 wk	Overweight	RCT	17 (51)	20–50	Sedentary	Laboratory-based	19	Fair
[29]	No	16 wk	Overweight	RCT	17 (51)	20–50	Sedentary	Laboratory-based	19	Fair
[53]	No	12 wk	No data	NRCT	10 (26)	(25 ± 2.5)	No exercise habit	Laboratory-based	15	Low
[32]	No	10 wk	Overweight	RCD	39	≥55	Sedentary	Laboratory-based	21	Fair
[55]	No	7 wk	No data	RCT	9 (24)	18–30	Untrained	Laboratory-based	17	Fair
[35]	No	5 d	Overweight	RCD	23	35–65 (48.2 ± 7.9)	Sedentary	Laboratory-based	21	Fair
[57]^b^	No	4 d	Normal	RCD	18	(21 ± 2)	Physically inactive	Laboratory-based	18	Fair
[63]^a^	No	~238 min	No data	RCD	13	(23.8 ± 0.9)	Physically active	Laboratory-based	19	Fair
[54]^b^	No	~214.5 min	Normal	RCD	9	(24.0 ± 4.0)	Recreationally active	Laboratory-based	17	Fair
[49]^a^	No	120 min	No data	RCD	12	(25.8 ± 1.2)	Regular physical activity	Laboratory-based	14	Low
[50]	No	120 min	Normal	Case series	22	18–23	Regular structured running	Laboratory-based	13	Low
[44]	No	120 min	No data	RCD	6	(25 ± 2)	Moderately trained	Laboratory-based	15	Low
[47]^a^	No	90 min	Normal	RCD	12	(29.5 ± 6.2 male; 24.3 ± 1.4 female)	Recreationally active	Laboratory-based	15	Low
[46]	No	90 min	No data	RCD	12	(28.2 ± 1.5)	Recreationally active	Laboratory-based	15	Low
[51]	No	45 min	Normal	URCT	22	(23.22 ± 2.88)	Runners (<5 miles/wk)	Laboratory-based	15	Low
[37]	No	40 min	Overweight	RCD	12	(46.2 ± 1.1)	Sedentary	Laboratory-based	20	Fair
[45]	No	35 min	Normal	RCD	14	(25.4 ± 1.8)	Endurance trained	Laboratory-based	16	Fair
[40]^b^	No	28 min	Overweight	RCD	10	(24 ± 3)	No data	Laboratory-based	18	Fair
[38]^b^	No	28 min	Obese	RCD	19	45–65	Physically inactive	Laboratory-based	24	High
[39]^b^	No	28 min	Obese	RCD	19	45–65	Physically inactive	Laboratory-based	22	High
[52]	No	15 min	Normal	RCD	6	22–39 (32 ± 4)	Athletes	Laboratory-based	15	Low
[48]	No	5 min	No data	RCD	6	(23.5 ± 0.9)	Sedentary	Laboratory-based	14	Low
[31]	HPN	12 wk	Obese	RCT	20 (40)	45–65	Sedentary	Free-living	20	Fair
[41]	DM2	12 wk	No data	RCT	29 (59)	(60 ± 10)	No data	Laboratory-based	14	Low
[42]	CHF	8 wk	No data	RCT	7 (21)	(58 ± 3)	Habitually active	Laboratory-based	16	Fair
[33]	GDM	6 wk	Obese	Case control	10 (30)	(33.4 ± 3.3)	No data	Free-living	16	Fair
[34]	Colon Cancer	2 wk	Overweight	RCT	10 (23)	59–67	No data	Laboratory-based	14	Low
[36]^a^	MS	≈103 min	Obese	RCD	14	(43 ± 9)	Physically inactive	Laboratory-based	16	Fair
[43]	HIV infected	60 min	No data	RCT	11 (38)	(42.9 ± 7.5)	Exercise-naive	Laboratory-based	18	Fair

BMI: body mass index; DM2: diabetes mellitus type 2; GDM: gestational diabetes mellitus; HIV: human immunodeficiency virus; HPN: hypertension; LIPA: light intensity physical activity; min: minute; mo: month; MS: metabolic syndrome; NRCT: nonrandomized controlled trial; RCD: randomized cross-over design; RCT: randomized controlled trial; URCT: uncontrolled randomized clinical trial; wk: week. Physical inactivity is defined as not meeting at least 150 minutes of moderate intensity physical activity or 75 minutes of vigorous intensity physical activity. ^a^Compared to a control group with no prescribed activity. ^b^Compared to a sitting group. ^c^Disease status and baseline activity level (using the terminology of the primary study) presented were as per inclusion criteria.

**Table 2:** Summary of studies examining the effect of LIPA on CVD risk factors and CVD-related markers.

Marker	Summary coding of studies involving physically inactive population		Summary coding of studies involving populations with a medical condition		Summary coding of studies involving healthy population		Summary coding of all included studies	
CVD risk factors	*n*/*N* (%)^a,b,c^	Effect (0/−/+/?)^d^	*n*/*N* (%)^a,b,c^	Effect (0/−/+/?)^d^	*n*/*N* (%)^a,b,c^	Effect (0/−/+/?)^d^	*n*/*N* (%)^a,b,c^	Effect (0/−/+/?)^d^

Body mass	0/4 (0%)	No (00)	0/0 (0%)	NA	0/4 (0%)	No (00)	0/5 (0%)	No (00)
WC	0/3 (0%)	No (0)	0/2 (0%)	No (0)	0/0 (0%)	NA	0/4 (0%)	No (00)
BMI	0/3 (0%)	No (0)	0/2 (0%)	No (0)	0/0 (0%)	NA	0/4 (0%)	No (00)
% BF	0/2 (0%)	No (0)	0/0 (0%)	NA	0/0 (0%)	NA	0/2 (0%)	No (0)
Systolic BP	3/5 (60%)	Positive (++)	2/3 (67%)	Positive (+)	0/5 (0%)	No (00)	3/9 (33%)	No (00)
Diastolic BP	2/5 (40%)	Inconsistent (??)	2/3 (67%)	Positive (+)	0/5 (0%)	No (00)	2/9 (22%)	No (00)
Glucose	1/8 (12%)	No (00)	1/3 (33%)	No (0)	0/8 (0%)	No (00)	3/16 (19%)	No (00)
Insulin	1/8 (12%)	No (00)	0/1 (0%)	NA	0/8 (0%)	No (00)	1/13 (8%)	No (00)
Hba1C	0/1 (0%)	NA	0/1 (0%)	NA	0/0 (0%)	NA	0/2 (0%)	No (0)
Total cholesterol	1/5 (20%)	No (00)	0/0 (0%)	NA	1/6 (17%)	No (00)	2/11(18%)	No (00)
HDL cholesterol	0/7 (0%)	No (00)	0/1 (0%)	NA	1/8 (12%)	No (00)	1/13 (8%)	No (00)
LDL cholesterol	0/4 (0%)	No (00)	0/0 (0%)	NA	0/3 (0%)	No (0)	0/6 (0%)	No (00)
Triglycerides	2/8 (25%)	No (00)	0/1 (0%)	NA	4/7 (57%)	Inconsistent (??)	5/13 (38%)	Inconsistent (??)

CVD-related markers	*n*/*N* (%)^a,b,c^	Effect (0/−/+/?)^d^	*n*/*N* (%)^a,b,c^	Effect (0/−/+/?)^d^	*n*/*N* (%)^a,b,c^	Effect (0/−/+/?)^d^	*n*/*N* (%)^a,b,c^	Effect (0/−/+/?)^d^

VO_2_ max	2/5 (40%)	Inconsistent (??)	0/1 (0%)	NA	1/2 (50%)	Inconsistent (?)	3/8 (38%)	Inconsistent (??)
Resting HR	1/4 (25%)	No (00)	0/1 (0%)	NA	0/3 (0%)	No (0)	1/5 (20%)	No (00)
CRP	0/2 (0%)	No (0)	0/0 (0%)	NA	0/0 (0%)	NA	0/2 (0%)	No (0)
Interleukin-6	0/3 (0%)	No (0)	0/2 (0%)	No (0%)	0/0 (0%)	NA	0/4 (0%)	No (00)
TNF-alpha	0/1 (0%)	NA	0/1 (0%)	NA	0/0 (0%)	NA	0/2 (0%)	No (0)

^a^ *n* = number of studies reporting difference in the expected direction.

^b^ *N* = number of identified studies of interest.

^c^(%) = percentage of studies reporting differences in the expected direction.

^d^Summary effect. No effect (0): 0–33% of studies reported significant differences; inconsistent (?): 34–59% of studies reported significant differences; positive (+) or negative (−) effect: 60–100% of studies demonstrated significant differences; ≥4 studies: positive (++), negative (−−), no effect (00), and inconsistent findings (??).

CVD markers (waist-to-hip ratio, heart rate maximal, and tumor necrosis factor receptor 2) with only one study demonstrating the effect of light intensity activity were excluded in this summary table.

BF: body fat; BMI: body mass index; BP: blood pressure; CRP: C-reactive protein; CVD: cardiovascular disease; Hba1c: glycosylated hemoglobin; HDL: high-density lipoprotein; HR: heart rate; LDL; low-intensity lipoprotein; NA: not applicable; TNF: tumor necrosis factor; VO_2_ max: maximal oxygen uptake; WC: waist circumference.
