# Supplementary material for: Effects of Light Intensity Activity on CVD Risk Factors: A Systematic Review of Intervention Studies
Source: Biomed Res Int. 2015 Oct 12;2015:596367. doi: 10.1155/2015/596367 (PMC4620294; doi:10.1155/2015/596367)
Supplement: Supplementary file 1 — The Supplementary Material files provide the search criteria used in the review (Supplementary Table 1), detailed description of included studies (Supplementary Table 2), and the summary effects of LIPA on CVD risk factors and CVD-related markers reported per study (Supplementary Table 3). [file 596367.f1.pdf]

Table 1. Review search criteria.

**Search terms**	(light-intensity training OR low-intensity training OR light-intensity exercise OR low-intensity exercise OR light-intensity activity OR low-intensity activity OR light-intensity physical activity OR low-intensity physical activity OR light-intensity walking OR light-intensity lifestyle OR light intensity training OR low intensity training OR mild intensity training OR light intensity exercise OR low intensity exercise OR mild intensity exercise OR light intensity activity OR low intensity activity OR mild intensity activity OR light intensity physical activity OR low intensity physical activity OR light intensity walking OR mild intensity walking OR light intensity lifestyle OR LIPA) AND (fasting plasma glucose OR glycosylated hemoglobin OR HbA1c OR triglycerides OR insulin OR total cholesterol OR LDL cholesterol OR HDL cholesterol OR CRP OR C-reactive protein OR IL-6 OR interleukin-6 OR TNF-alpha OR TNF receptor-1 OR TNFR1 OR TNF receptor-2 OR TNFR2 OR "body mass index" OR BMI OR waist circumference OR hip circumference OR waist-to-hip ratio OR resting heart rate OR percent body fat OR lean body mass OR resting blood pressure OR maximum heart rate OR VO_2 max) AND Humans[MeSH] AND Adult[MeSH] AND English[lang]
**Publication types**	clinical trials, randomized controlled trials, controlled clinical trials, and comparative studies

Table 2. Detailed description of included studies.

Study	Duration of Intervention	Study Design	Baseline Activity Level[a]	Activity Level Intensity (used criteria)	Modality	Duration and Frequency	Method of activity measurement	Compliance	Mean change in activity
Stevenson & Topp 1990 [56]	9 mo	RCT	No data	30-40% HRR	Stretching and calisthenics, stationary cycling, slow walking	30 min 3x/wk	HR monitor	No data	No data
Keller & Trevino 2001 [26]	24 wk	RCT	Sedentary (exercising <30min/wk)	50% target HR	Walking at 50% of target HR	30 min 3x/wk	Pedometer	88%	No data
Krause et al. 2014 [30]	16 wk	RCT	Sedentary (not engaged in any regular physical activity for the last 6 mo)	30-40% VO_2 max	Outdoor walking	30 min 3x/week	HR monitor	92% (44 ± 7 of 48 walking sessions)	No data
Cox et al. 2004 [28]	16 wk	RCT	Sedentary (<2 30-min sessions of vigorous exercise/wk)	17-24% HRR (slow flexibility exercises)	Stationary cycling, slow flexibility exercises and slow walking	30 min 3x/wk	Ergometer	No data	No data
Cox et al. 2003 [27]	16 wk	RCT	Sedentary (<2 30-min sessions of vigorous exercise/wk)	17-24% HRR (slow flexibility exercises)	Stationary cycling, slow flexibility exercises and slow walking	30 min 3x/wk	Ergometer	100%	No data
Cox et al.1996 [29]	16 wk	RCT	Sedentary (<2 30-min sessions of vigorous exercise/wk)	17-24% HRR (slow flexibility exercises)	Stationary cycling, slow flexibility exercises and slow walking	30 min 3x/wk	Ergometer	100%	No data

Table 2 (continued)

Study	Duration of Intervention	Study Design	Baseline Activity Level[a]	Activity Level Intensity (used criteria)	Modality	Duration and Frequency	Method of activity measurement	Compliance	Mean change in activity
Goldie et al. 2013[31]	12 wk	RCT	Sedentary (fewer than 3x/wk of aerobic exercise)	< 40% HRR	Home walking	0.8km/d for 2 wk increased every wk (400m/d) to a max of 4.8km/d 3-5x/wk	Self-reported	81%	No data
Goto et al. 2003 [53]	12 wk	NRCT	No exercise habit[b]	25% VO_2 max	Stationary cycling	30 min 5x-7x/wk	Ergometer	100%	No data
Skoro-Kondza et al. 2009 [41]	12 wk	RCT	No data	2.5 METs	Yoga	90 min 2x/wk	No data	No data	No data
Cornelissen et al. 2009 [32]	10 wk	RCD	Sedentary (≤30min of heavy exercise/wk)	33% HRR	Treadmill walking, stationary cycling, stepping	60 min 3x/wk	HR monitor	90.8%	No data
Gordon et al. 1996 [42]	8 wk	RCT	Habitually active[b]	35% VO_2 max	Stationary cycling	15 min 3x/wk	Ergometer	100%	No data
Van Den Berg et al. 2010 [55]	7 wk	RCT	Untrained[b]	30% HRR	Hand rim wheelchair training on a motor-driven treadmill	30 min 3x/wk	HR monitor	100%	No data
Davenport et al. 2008 [33]	6 wk	Case Control Study	No data	30% HRR	Walking at 30% HRR	40 min 3-4x/wk	HR monitor	No data	No data
Allgayer et al. 2004 [34]	2 wk	RCT	No data	30-40% VO_2 max	Stationary cycling	40 min/d	Ergometer	No data	No data

Table 2 (continued)

**Study**	**Duration of Intervention**	**Study Design**	**Baseline Activity Level**[a]	**Activity Level Intensity (used criteria)**	**Modality**	**Duration and Frequency**	**Method of activity measurement**	**Compliance**	**Mean change in activity**
Thorp et al. 2014 [35]	5 d	RCD	Sedentary (desk-bound occupation)	< 3 METs (Interchanging seated and standing posture every 30 min for 8hrs/d)	Standing in position with light ambulatory movement permitted	30 min standing every 30 min 8x/day	Activity monitor	97-98%	Sitting group (min/d): 468.1 ± 1.3 sitting; 3.2 ± 0.4 stepping; 7.9 ± 0.8 standing LIPA group (min/d): 232.0 ± 1.4 sitting; 5.6 ± 0.4 stepping; 242.5 ± 1.5 standing
Duvivier et al. 2013 [57]	4 d	RCD	Physical exercise less than 1hr/wk	< 3 METs	Leisure walking (4 hr) and standing (2 hr)	4 hr walking and 2 hr standing/d	Activity monitor	100%	Sitting group: 13.6 ± 1.2 hr/d sitting; 0.99 ± 0.5 hr/d standing; 4324 ± 1485 steps/d LIPA group: 7.4 ± 1.3 hr/d sitting; 3.08 ± 0.88 hr/d standing; 27590 ± 3724 steps/d

Table 2 (continued)

Study	Duration of Intervention	Study Design	Baseline Activity Level[a]	Activity Level Intensity (used criteria)	Modality	Duration and Frequency	Method of activity measurement	Compliance	Mean change in activity
Katsanos et al. 2004 [58]	237.5 ± 9 min	RCD	Physically active[b]	25% VO_2 max	Treadmill walking	237.5 ± 9 min single bout	Expired gas measurement	100%	Pre-Intervention: No data Post-Intervention: 237.5 ± 9.0 min single bout walking
Kim et al. 2014 [54]	214.5 ± 28.0 min	RCD	Recreationally active[b]	25% VO_2 max	Treadmill walking	214.5 ± 28.0 min divided into 9 intermittent bouts	Expired gas measurement	100%	Sitting group (min/d): 476 ± 36 sitting; 6.8 ± 3 walking LIPA group (min/d): 226 ± 55 sitting; 213.9 ± 34 walking
Aldred, Perry & Hardman 1994 [49]	120 min	RCD	Regular physical activity[b]	30.9% ± 1.6% VO_2 max	Treadmill walking	120 min single bout	Expired gas measurement	100%	No data
Pay et al. 1992 [50]	120 min	Case Series	Endurance-trained (regular structured program running)	30% VO_2 max (29.8 ± 3.9%)	Treadmill walking	120 min single bout	Expired gas measurement	100%	No data
Horowitz et al. 1999 [44]	120 min	RCD	Moderately trained[b]	25% VO_2 peak	Stationary cycling	120 min single bout	Expired gas measurement	100%	No data

Table 2 (continued)

Study	Duration of Intervention	Study Design	Baseline Activity Level[a]	Activity Level Intensity (used criteria)	Modality	Duration and Frequency	Method of activity measurement	Compliance	Mean change in activity
Mestek et al. 2008 [36]	102.8 ± 4.3 min	RCD	Physically inactive (< 150 min of moderate intensity physical activity/wk for at least 3 mo)	35-45% VO_2 max (38.6 ± 2.2)	Treadmill walking	102.8 ± 4.3 min single bout	Expired gas measurement	100%	Pre-Intervention: No data Post-Intervention: 102.8 ± 4.3 min single bout walking
Tsetsonis & Hardman 1996 [47]	90 min	RCD	Recreationally active but not well-trained[b]	30% VO_2 max	Treadmill walking	90 min single bout	Expired gas measurement	100%	No data
Tsetsonis & Hardman 1995 [46]	90 min	RCD	Recreationally active not trained[b]	30% VO_2 max	Treadmill walking	90 min single bout	Expired gas measurement	100%	No data
Dudgeon et al. 2010 [43]	60 min	RCT	Exercise-naïve (no involvement in a structured exercise program)	50% HR max	Treadmill walking and/or stationary cycling	60 min single bout	Ergometer	100%	No data
Hughes et al. 1990 [51]	45 min	URCT	Novice runners (running <5miles/wk)	20% below VT (=30% VO_2R or HRR)	Treadmill walking	45 min single bout	Expired gas measurement	100%	No data
Mendham et al. 2011 [37]	40 min	RCD	Sedentary (not involved in >1 regular exercise session/wk for >20min)	30% VO_2 max	Stationary cycling	40 min single bout	HR monitor	100%	No data

Table 2 (continued)

Study	Duration of Intervention	Study Design	Baseline Activity Level[a]	Activity Level Intensity (used criteria)	Modality	Duration and Frequency	Method of activity measurement	Compliance	Mean change in activity
Fujimoto et al. 2003 [45]	35 min	RCD	Endurance trained (regular participation in endurance training /competitive sports)	30% VO_2 max	Stationary cycling	35 min single bout	Ergometer	100%	No data
Bailey & Locke 2014 [40]	28 min	RCD	No data	2.9 METs (treadmill walking at 3.2 km/hr)	Treadmill walking	2-min bout every 20 min (28 min) over 5 hr treatment period	Borg RPE scale	100%	No data
Dunstan et al. 2012 [38]	28 min	RCD	Physically inactive (< 150 min of moderate intensity physical activity/wk for at least 3 mo)	2.9 METs (treadmill walking at 3.2 km/hr)	Treadmill walking	2-min bout every 20 min (28 min) over 5 hr treatment period	Activity monitor	100%	No data
Larsen et al. 2014 [39]	28 min	RCD	Physically inactive (< 150 min of moderate intensity physical activity/wk for at least 3 mo)	2.9 METs (treadmill walking at 3.2 km/hr)	Treadmill walking	2-min bout every 20 min (28 min) over 5 hr treatment period	Activity monitor	100%	No data
Wittert et al. 1991 [52]	15 min	RCD	Athletes[b]	20% $V0_2$ max	Treadmill walking	15 min single bout	No data	100%	No data
Perini et al. 1989 [48]	5 min	RCD	Sedentary (sedentary not defined)	15-33% VO_2 max (21.5% ± 2.8%)	Stationary cycling	5 min single bout	Ergometer	100%	No data

Abbreviations: d, day; ECG, electrocardiogram; HR, heart rate; hr, hour; HRR, heart rate reserve; max, maximum; METs, metabolic equivalents; min, minute; mo, month; NRCT, non-randomized controlled trial; RCD, randomized cross-over design; RCT, randomized controlled trial; URCT, uncontrolled randomized clinical trial; VO_2 max, maximal oxygen consumption; VO_2 peak, peak rate of oxygen consumption; VO_2R, oxygen consumption reserve; VT, ventilatory threshold; wk, week. [a]Described using the terminology of the primary study. [b]Level of activity not clearly described in the study. Compliance refers to the number of activity sessions completed by the participants (expressed in %).

Table 3. Summary of effects of LIPA on CVD risk factors and CVD-related markers per study, sorted by disease status and duration of intervention.

Study Reference	Duration of Intervention	BMI >25	Disease Status	Body Composition					Cardiorespiratory Fitness					Glucose Metabolism			Blood Lipids				Inflammatory			
				BM	WC	BMI	WHR	%BF	RHR	HRM	VO2	SBP	DBP	GLU	INS	Hba1c	TOT	HDL	LDL	TRI	CRP	IL6	TNFα	TNFR2
[56]	9 mo	ND	No	n/a	n/a	n/a	n/a	n/a	↔	n/a	↑	↔	↔	n/a	n/a	n/a	n/a	n/a	n/a	n/a	n/a	n/a	n/a	n/a
[26][a]	24 wk	Yes	No	↔	n/a	↔	↔	n/a	n/a	n/a	n/a	n/a	n/a	n/a	n/a	n/a	↓	↔	↔	↔	n/a	n/a	n/a	n/a
[30]	16 wk	Yes	No	n/a	n/a	n/a	n/a	↔	n/a	n/a	↔	↔	↔	↔	↔	↔	↔	↔	↔	↔	↔	↔	↔	n/a
[28]	16 wk	Yes	No	n/a	n/a	n/a	n/a	n/a	n/a	n/a	↔	n/a	n/a	n/a	n/a	n/a	n/a	n/a	n/a	n/a	n/a	n/a	n/a	n/a
[27]	16 wk	Yes	No	n/a	n/a	n/a	n/a	n/a	n/a	↔	↑	n/a	n/a	n/a	n/a	n/a	n/a	n/a	n/a	n/a	n/a	n/a	n/a	n/a
[29]	16 wk	Yes	No	↔	n/a	n/a	n/a	n/a	n/a	n/a	↔	n/a	n/a	n/a	n/a	n/a	n/a	n/a	n/a	n/a	n/a	n/a	n/a	n/a
[53]	12 wk	ND	No	↔	n/a	n/a	n/a	n/a	↔	n/a	n/a	↔	↔	↔	↔	n/a	↔	↔	n/a	↔	n/a	n/a	n/a	n/a
[32]	10 wk	Yes	No	n/a	↔	↔	n/a	↔	↓	n/a	↑	↓	↔	↔	↔	n/a	↔	↔	↔	↔	n/a	n/a	n/a	n/a
[55]	7 wk	ND	No	↔	n/a	n/a	n/a	n/a	n/a	n/a	↔	n/a	n/a	n/a	n/a	n/a	n/a	n/a	n/a	n/a	n/a	n/a	n/a	n/a
[35]	5 d	Yes	No	↔	↔	n/a	n/a	n/a	n/a	n/a	n/a	n/a	n/a	↔	↔	n/a	n/a	n/a	n/a	↔	n/a	n/a	n/a	n/a
[57][b]	4 d	No	No	n/a	n/a	n/a	n/a	n/a	n.a	n/a	n/a	n/a	n/a	↔	↔	n/a	↔	↔	↔	↓	n/a	n/a	n/a	n/a
[58][a]	≈238 min	ND	No	n/a	n/a	n/a	n/a	n/a	n/a	n/a	n/a	n/a	n/a	↔	↔	n/a	n/a	↔	n/a	↔	n/a	n/a	n/a	n/a
[54][b]	≈214 min	No	No	n/a	n/a	n/a	n/a	n/a	n/a	n/a	n/a	n/a	n/a	↔	↔	n/a	n/a	n/a	n/a	↓	n/a	n/a	n/a	n/a
[49][a]	120 min	ND	No	n/a	n/a	n/a	n/a	n/a	n/a	n/a	n/a	n/a	n/a	↔	↔	n/a	↔	↔	n/a	↓	n/a	n/a	n/a	n/a
[50]	120 min	No	No	n/a	n/a	n/a	n/a	n/a	n/a	n/a	n/a	n/a	n/a	n/a	n/a	n/a	↑	↑	n/a	n/a	n/a	n/a	n/a	n/a
[44]	120 min	ND	No	n/a	n/a	n/a	n/a	n/a	n/a	n/a	n/a	n/a	n/a	↔	↔	n/a	n/a	n/a	n/a	n/a	n/a	n/a	n/a	n/a
[47][a]	90 min	No	No	n/a	n/a	n/a	n/a	n/a	n/a	n/a	n/a	n/a	n/a	↔	↔	n/a	↔	↔	n/a	n/a	n/a	n/a	n/a	n/a
[46]	90 min	ND	No	n/a	n/a	n/a	n/a	n/a	n/a	n/a	n/a	n/a	n/a	n/a	n/a	n/a	↓	↔	↔	↓	n/a	n/a	n/a	n/a
[51]	45 min	No	No	n/a	n/a	n/a	n/a	n/a	n/a	n/a	n/a	n/a	n/a	n/a	n/a	n/a	↔	↔	↔	↔	n/a	n/a	n/a	n/a
[37]	40 min	Yes	No	n/a	n/a	n/a	n/a	n/a	n/a	n/a	n/a	n/a	n/a	n/a	n/a	n/a	n/a	n/a	n/a	n/a	↔	↔	n/a	n/a
[45]	35 min	No	No	n/a	n/a	n/a	n/a	n/a	n/a	n/a	n/a	n/a	n/a	↔	↔	n/a	n/a	n/a	n/a	n/a	n/a	n/a	n/a	n/a
[40][b]	28 min	Yes	No	n/a	n/a	n/a	n/a	n/a	n/a	n/a	n/a	↔	↔	↓	n/a	n/a	↔	↔	n/a	↔	n/a	n/a	n/a	n/a
[38][b]	28 min	Yes	No	n/a	n/a	n/a	n/a	n/a	n/a	n/a	n/a	n/a	n/a	↓	↓	n/a	n/a	n/a	n/a	n/a	n/a	n/a	n/a	n/a
[39][b]	28 min	Yes	No	n/a	n/a	n/a	n/a	n/a	n/a	n/a	n/a	↓	↓	n/a	n/a	n/a	n/a	n/a	n/a	n/a	n/a	n/a	n/a	n/a
[52]	15 min	No	No	n/a	n/a	n/a	n/a	n/a	n/a	n/a	n/a	↔	↔	n/a	n/a	n/a	n/a	n/a	n/a	n/a	n/a	n/a	n/a	n/a
[48]	5 min	ND	No	n/a	n/a	n/a	n/a	n/a	↔	n/a	n/a	n/a	n/a	n/a	n/a	n/a	n/a	n/a	n/a	n/a	n/a	n/a	n/a	n/a
[31]	12 wk	Yes	HPN	n/a	↔	↔	n/a	n/a	↔	n/a	n/a	↓	↓	n/a	n/a	n/a	n/a	n/a	n/a	n/a	n/a	n/a	n/a	n/a
[41]	12 wk	ND	DM2	n/a	↔	↔	n/a	n/a	n/a	n/a	n/a	↔	↔	↔	n/a	↔	n/a	n/a	n/a	n/a	n/a	n/a	n/a	n/a
[42]	8 wk	ND	CHF	n/a	n/a	n/a	n/a	n/a	n/a	n/a	↔	n/a	n/a	n/a	n/a	n/a	n/a	n/a	n/a	n/a	n/a	n/a	n/a	n/a
[33]	6 wk	Yes	GDM	n/a	n/a	n/a	n/a	n/a	n/a	n/a	n/a	n/a	n/a	↓	n/a	n/a	n/a	n/a	n/a	n/a	n/a	n/a	n/a	n/a
[34]	2 wk	Yes	Colon Cancer	n/a	n/a	n/a	n/a	n/a	n/a	n/a	n/a	n/a	n/a	n/a	n/a	n/a	n/a	n/a	n/a	n/a	n/a	↔	↔	n/a
[36][a]	≈103 min	Yes	MS	n/a	n/a	n/a	n/a	n/a	n/a	n/a	n/a	n/a	n/a	↔	↔	n/a	n/a	↔	n/a	↓	n/a	n/a	n/a	n/a
[43]	60 min	ND	HIV	n/a	n/a	n/a	n/a	n/a	n/a	n/a	n/a	n/a	n/a	n/a	n/a	n/a	n/a	n/a	n/a	n/a	n/a	↔	n/a	↔
Number of studies:				5	4	4	1	2	5	1	8	9	9	16	13	2	11	13	6	13	2	4	2	1

Abbreviations: % BF, body fat percentage; BM, body mass; BMI, body mass index; CHF, chronic heart failure; CRP, C-reactive protein; d, day; DBP, diastolic blood pressure; DM2, type 2 diabetes mellitus; GLU, glucose; GDM, gestational diabetes mellitus; HbA1c, glycosylated hemoglobin; HDL, high-density lipoprotein; HIV, human immunodeficiency virus; HPN, essential hypertension; HRM, heart rate maximal; INS, insulin; IL-6, interleukin-6; LDL, low-density lipoprotein; min, minute; MS, metabolic syndrome; ND, no data; RHR, resting heart rate; SBP, systolic blood pressure; TNF-α, tumor necrosis factor-alpha; TNFR2, tumor necrosis factor receptor 2; TOT, total cholesterol; TRI, triglycerides; VO_2 max, maximal oxygen uptake; WC, waist circumference; WHR, waist-to-hip ratio; wk, week; ↔ no statistically significant change; ↑statistically significant increase; ↓statistically significant increase; n/a, not applicable. [a]compared to a control group with no prescribed activity; [b]compared to sitting group. Only outcomes statistically compared to baseline or a control group with no prescribed activity was used in the study.
